# Retraction: Melatonin attenuates dysregulation of the circadian clock pathway in mice with CCl4-induced fibrosis and human hepatic stellate cells

**DOI:** 10.3389/fphar.2023.1252194

**Published:** 2023-07-07

**Authors:** Frontiers Editorial Office

Following publication, concerns were raised regarding the integrity of the images in the published figures. Image duplication concerns were identified in **Figures 1A, 2C, 6B**. The authors failed to provide a satisfactory explanation during the investigation, which was conducted in accordance with Frontiers’ policies. Given the concerns about the validity of the data, and the lack of raw data, the editors no longer have confidence in the findings presented in the article.

This retraction was approved by the Chief Editors of Frontiers in Pharmacology and the Chief Executive Editor of Frontiers. The authors agree to this retraction.

